# Cx47 fine-tunes the handling of serum lipids but is dispensable for lymphatic vascular function

**DOI:** 10.1371/journal.pone.0181476

**Published:** 2017-07-21

**Authors:** Merlijn J. Meens, Issa Kutkut, Viviane Rochemont, Juan Dubrot, Fouad R. Kaladji, Amélie Sabine, Oliver Lyons, Stefanie Hendrikx, Jeremiah Bernier-Latmani, Friedemann Kiefer, Alberto Smith, Stéphanie Hugues, Tatiana V. Petrova, Brenda R. Kwak

**Affiliations:** 1 Department of Pathology and Immunology, University of Geneva, Geneva, Switzerland; 2 Department of Fundamental Oncology, Centre Hospitalier Universitaire Vaudois (CHUV), University of Lausanne, Lausanne, Switzerland; 3 Division of Experimental Pathology, Institute of Pathology, CHUV, Lausanne, Switzerland; 4 Academic Department of Vascular Surgery, Cardiovascular Division, King's College London, BHF Centre of Research Excellence & NIHR Biomedical Research Centre at King's Health Partners, St Thomas' Hospital, London, United Kingdom; 5 Mammalian Cell Signalling Laboratory, Department of Vascular Cell Biology, Max Planck Institute for Molecular Biomedicine, Münster, Germany; 6 Swiss Institute for Experimental Cancer Research, School of Life Sciences, Swiss Federal Institute of Technology Lausanne, Lausanne, Switzerland; 7 Department of Medical Specialties – Cardiology, University of Geneva, Geneva, Switzerland; Ludwig-Maximilians-Universitat Munchen, GERMANY

## Abstract

Mutations in the gap junction protein connexin47 (Cx47) are associated with lymphedema. However, the role of Cx47 in lymphatic pathophysiology is unknown. We demonstrate that Cx47 is expressed in lymphatic endothelial cells by whole-mount immunostaining and qPCR. To determine if Cx47 plays a role in lymphatic vessel function we analysed *Cx47*^*-/-*^ mice. Cx47-deficiency did not affect lymphatic contractility (contractile amplitude or frequency) or lymphatic morphology (vessel diameter or number of valves). Interstitial fluid drainage or dendritic cell migration through lymphatic vessels was also not affected by Cx47-deficiency. Cx47 is dispensable for long-chain fatty acid absorption from the gut but rather promotes serum lipid handling as prolonged elevated triglyceride levels were observed in Cx47-deficient mice after oral lipid tolerance tests. When crossed with Apolipoprotein E-deficient (*Apoe*^*-/-*^) mice, LDL-cholesterol was decreased in young *Cx47*^*-/-*^*Apoe*^*-/-*^ adults as compared to *Apoe*^*-/-*^ mice, which was inverted later in life. Finally, advanced atherosclerotic plaques in thoracic-abdominal aortas of 15 months-old mice tended to be larger in *Cx47*^*-/-*^*Apoe*^*-/-*^ mice. These plaques contained fewer macrophages but similar amounts of T lymphocytes, collagen and lipids than plaques of *Apoe*^*-/-*^ mice. In conclusion, Cx47 is expressed in lymphatic endothelium and seems modestly implicated in multiple aspects of lymphatic pathophysiology.

## Introduction

Although the incidence of lymphedema is still on the rise, proper treatment for this painful and crippling condition is unavailable. Lymphedema can be inherited or secondary, *i*.*e*. caused by medical treatment or trauma. Importantly, it has recently been shown that mutations in various genes, such as *FOXC2*, *VEGFR3*, *SOX18*, *GATA2* and *CCBE1*, predispose to lymphedema, hinting to involvement of proteins encoded by these genes in this disease [1]. Recently, the connexin protein family has been added to the repertoire of lymphedema-associated genes [2–5].

Connexins are transmembrane proteins that are expressed throughout the body. These proteins are inserted in cell membranes as hexamers (connexons) that can be composed of one or several types of connexins. In specific pathological conditions, connexons can function as hemi-channels allowing for diffusion of small soluble factors from the cytosol to the extracellular space or *vice versa*. More commonly, a connexon docks to a connexon expressed by an adjacent cell thereby forming a gap junction channel allowing for cytoplasmic exchange of ions and small metabolites between neighboring cells [6]. As such, gap junction channels are critically involved in direct cell-cell communication and synchronization of tissue responses [7, 8].

Several autosomal recessive mutations in *GJC2* (which encodes connexin47 (CX47)), have been associated with lymphedema [3–5]. These mutations result in amino acid changes in various domains of the CX47 protein; *i*.*e*. H19P (N-terminus), S48L (first extracellular loop), R125Q and G149S (intracellular loop), R260C (second extracellular loop) and P316L (C-terminus). The functional consequences of these mutations on CX47 channels are largely unknown. Like Cx37 and Cx43 [9, 10], Cx47 has been detected in subgroups of lymphatic endothelial cells (LECs), particularly in lymphatic valves, of young mice [10, 11]. It is now well established that both Cx37 and Cx43 play important roles during lymphatic valve development [9–11]. However, only sparse and fractional information about the expression and role of Cx47 in adult lymphatic vessels exists [12, 13]. Hence, the current study addressed possible involvement of Cx47 in lymphatic pathophysiology.

## Results

### Cx47 is expressed in LECs

**Fig 1:**
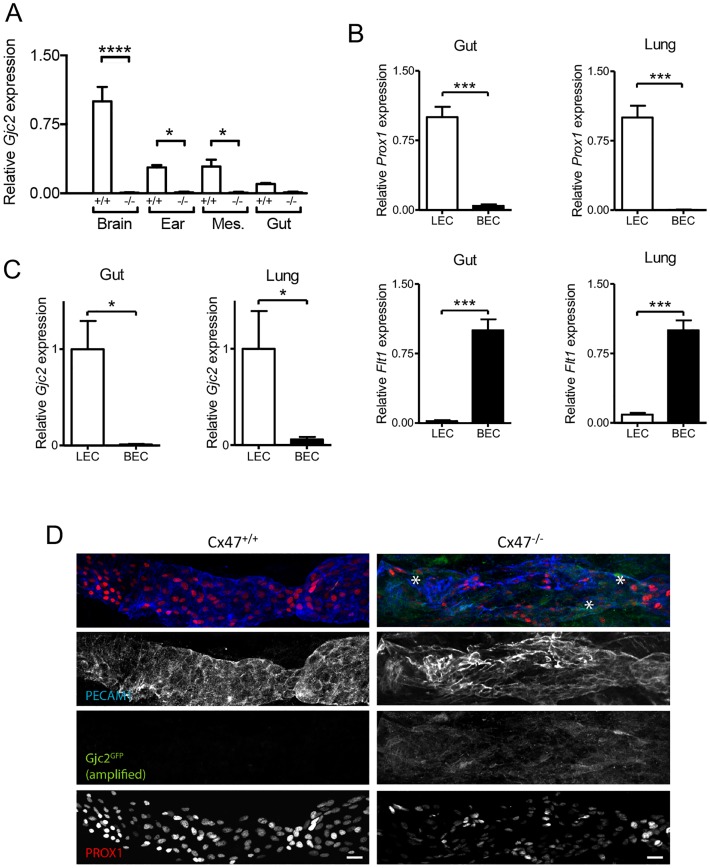
Cx47 is expressed by lymphatic endothelial cells. A) Expression of *Gjc2* in total mRNA isolated from the brain, ear, mesentery (Mes.) and gut from *Cx47*^*-/-*^ (black bars) or *Cx47*^*+/+*^ mice (white bars). N = 3. B) Expression of *Prox1* (upper panels) or *Flt1* (lower panels) in lymphatic endothelial cells (LECs; white bars) or blood endothelial cells (BECs; black bars) isolated from the gut (left panels) or the lung (right panels) of wild-type mice. C) Expression of *Gjc2* in LECs (white bars) or BECs (black bars) isolated from the gut (left panel) or lung (right panel) of wild-type mice. N = 6. D) Presence (asterisks) of eGFP in PROX1/PECAM1 positive LECs of Cx47^eGFP/eGFP^ but not Cx47^+/+^ mice. Scalebar equals 20 μm.

Initially Cx47 expression was thought to be restricted to oligodendrocytes in the central nervous system (CNS), where they contribute to myelination [14–16]. More recently, Cx47 has also been detected in lymphatic valve LECs of young mice [10, 11, 13]. Here, we studied expression of Cx47 in lymphatics of adult mice. Unfortunately, immunostainings with various commercially available Cx47 antibodies resulted in non-specific staining; *i*.*e*. prominent staining in tissues isolated from Cx47-deficient mice (data not shown). We therefore opted to use alternative approaches to demonstrate Cx47 expression. First, we isolated mRNA from different organs and assessed the presence of *Gjc2* (the gene encoding Cx47) by quantitative PCR (qPCR). As expected, *Gjc2* was expressed in the brain from Cx47-expressing (hereafter *Cx47*^*+/+*^) control mice whereas this was not the case for mRNA samples isolated from the brain of mice in which *eGFP* was knocked-in into the *Gjc2* locus (*Cx47*^*eGFP/eGFP*^; hereafter *Cx47*^*-/-*^) (Fig 1A). We also detected higher levels of *Gjc2* expression in mRNA samples isolated from the ear and mesentery of *Cx47*^*+/+*^ mice as compared to mRNA isolated from *Cx47*^*-/-*^ mice (Fig 1A). Moreover, this tended to be the case in mRNA isolated from the gut as well (Fig 1A). In order to identify the particular cell type expressing *Gjc2*, we isolated primary blood endothelial cells (BECs) and LECs from the gut and the lungs of wild-type (WT) mice. Initially, we confirmed the purity of these cell preparations by qPCR focusing on genes specific for BECs or LECs. LECs but not BECs expressed high levels of lymphatic endothelial cell specific *Prox1* (Fig 1B). On the contrary, in agreement with previous findings [13] BECs but not LECs expressed high levels of *Flt1* (which encodes VEGFR1; Fig 1B). Moreover, LECs but not BECs from the gut and the lung express *Gjc2* (Fig 1C). Finally, *Gjc2* promoter activity was assessed in 2 days-old *Cx47*^*eGFP/eGFP*^ mice by whole-mount confocal microscopy on lymphatic vessels in the hindlimb region using methods described previously for proximal femoral veins [17]. These experiments showed heterogeneous eGFP expression in PROX1/PECAM1 positive LECs in *Cx47*^*eGFP/eGFP*^ but not in *Cx47*^*+/+*^ mice (Fig 1D). Thus, *Gjc2* expression was observed in LECs of young as well as adult mice and Cx47 may thus play a role in lymphatic pathophysiology.

### Cx47 does not alter lymphatic morphology

**Fig 2:**
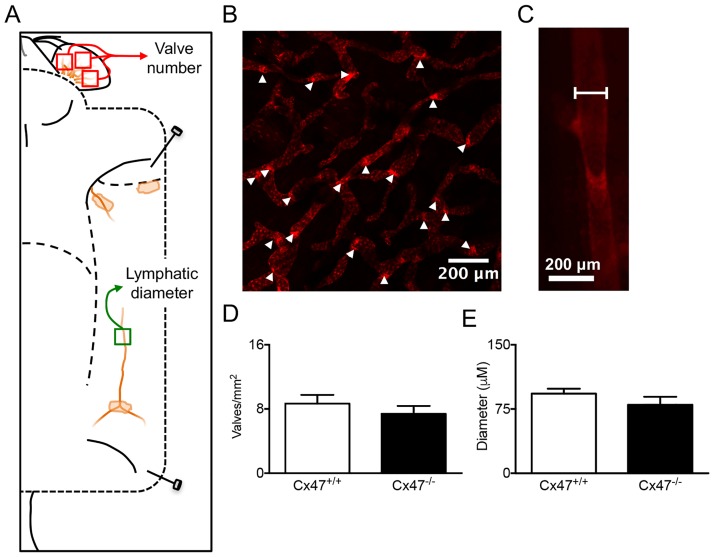
Normal lymphatic vessel organization in Cx47-deficient mice. A) Schematic illustration showing localization of mOrange2 positive lymphatic vessels in a *Cx47*^*+/+*^*Prox1*^*tg/+*^ mouse. As indicated (red boxes), the number of lymphatic valves was quantified in three separate images covering > 75% of the inner ear surface. Moreover, lymphatic diameter was quantified in the lymphatic vessel draining the inguinal lymph node (green box). B) Representative image showing lymphatic valves (arrowheads) in the inner ear of a *Cx47*^*+/+*^*Prox1*^*tg/+*^ mouse. C) Representative image showing the measurement of the lymphatic diameter in a *Cx47*^*+/+*^*Prox1*^*tg/+*^ mouse at equidistance between two valves. D) Quantification of the number of lymphatic valves in *Cx47*^*+/+*^*Prox1*^*tg*/+^ (white bar) and *Cx47*^*-/-*^*Prox1*^*tg/+*^ (black bar) mice. E) Quantification of the lymphatic diameter in *Cx47*^*+/+*^*Prox1*^*tg/+*^ (white bar) and *Cx47*^*-/-*^*Prox1*^*tg/+*^ (black bar) mice. N = 7.

Deficiency for other connexins (e.g. Cx37 and Cx43 [9–11]) has been linked to changes in lymphatic morphology. To explore whether Cx47-deficiency would alter lymphatic morphology we performed confocal microscopy on the dermis of ears isolated from *Cx47*^*-/-*^*Prox1*^*tg/+*^ or control (*Cx47*^*+/+*^*Prox1*^*tg/+*^) mice (Fig 2A) to quantify whether the number of lymphatic valves was affected by Cx47-deficiency (Fig 2B). Moreover, we used intravital microscopy on lymphatic collecting vessels of the same mice to quantify their diameter (Fig 2C). In line with an earlier study [18], these experiments showed that, contrary to Cx37- and Cx43-deficiency [9, 10], Cx47-deficiency does not affect the number of lymphatic valves (Fig 2D). In addition, they also indicated that lymphatic diameter is not affected by Cx47-deficiency (Fig 2E).

### Lymphatic Cx47 does not affect lymphatic contractility

**Fig 3:**
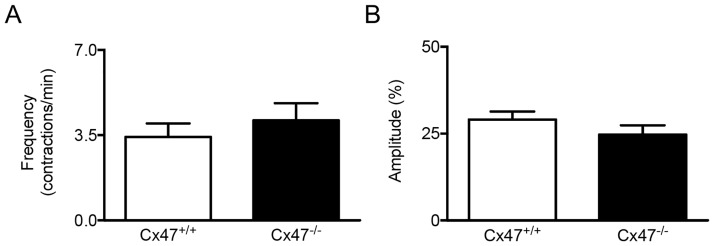
Lymphatic contractility is not regulated by Cx47. A/B) Quantification of the frequency (A) and amplitude (B) of lymphatic contractions in *Cx47*^*+/+*^*Prox1*^*tg/+*^ (white bars) and *Cx47*^*-/-*^*Prox1*^*tg/+*^ (black bars) mice. N = 7/11.

Previous studies have shown that lymphangions in collecting lymphatic vessels function as independent units; *i*.*e*. fluid flows in, this increases the intraluminal pressure resulting in the depolarization of a group of ‘pace-maker’ cells thereby creating an action potential that traverses the lymphangion and thus causes contraction and expulsion of the lymphatic fluid [19]. Interestingly, early studies have shown that synchronization of lymphatic tissue responses is disrupted in presence of chemical gap junction channel blockers such as heptanol or oleic acid [20, 21]. To investigate whether Cx47 is involved in coordinated contractility in lymphangions, we studied the amplitude and frequency of spontaneous contractions of the lymphangions in the lymphatic collecting vessel that drains the inguinal lymph node from *Cx47*^*-/-*^*Prox1*^*tg/+*^ or *Cx47*^*+/+*^*Prox1*^*tg/+*^ mice using intravital microscopy and video analysis. In line with expectations [19, 22], we observed rhythmic contractions with a frequency of approximately 3.5 contractions/minute (see S1 Movie for a typical example). This frequency was not affected by Cx47-deficiency (Fig 3A). Moreover, even though prominent contractile amplitudes were detected in lymphatic collecting vessels from *Cx47*^*+/+*^*Prox1*^*tg/+*^ mice, these were unaltered in collecting vessels from *Cx47*^*-/-*^*Prox1*^*tg/+*^ mice (Fig 3B). Thus, Cx47-deficiency in LECs does not directly affect the contractions of lymphatic vascular smooth muscle cells in Cx47-deficient mice.

### Lymphatic Cx47 does not contribute to drainage of lymphatic fluids

**Fig 4:**
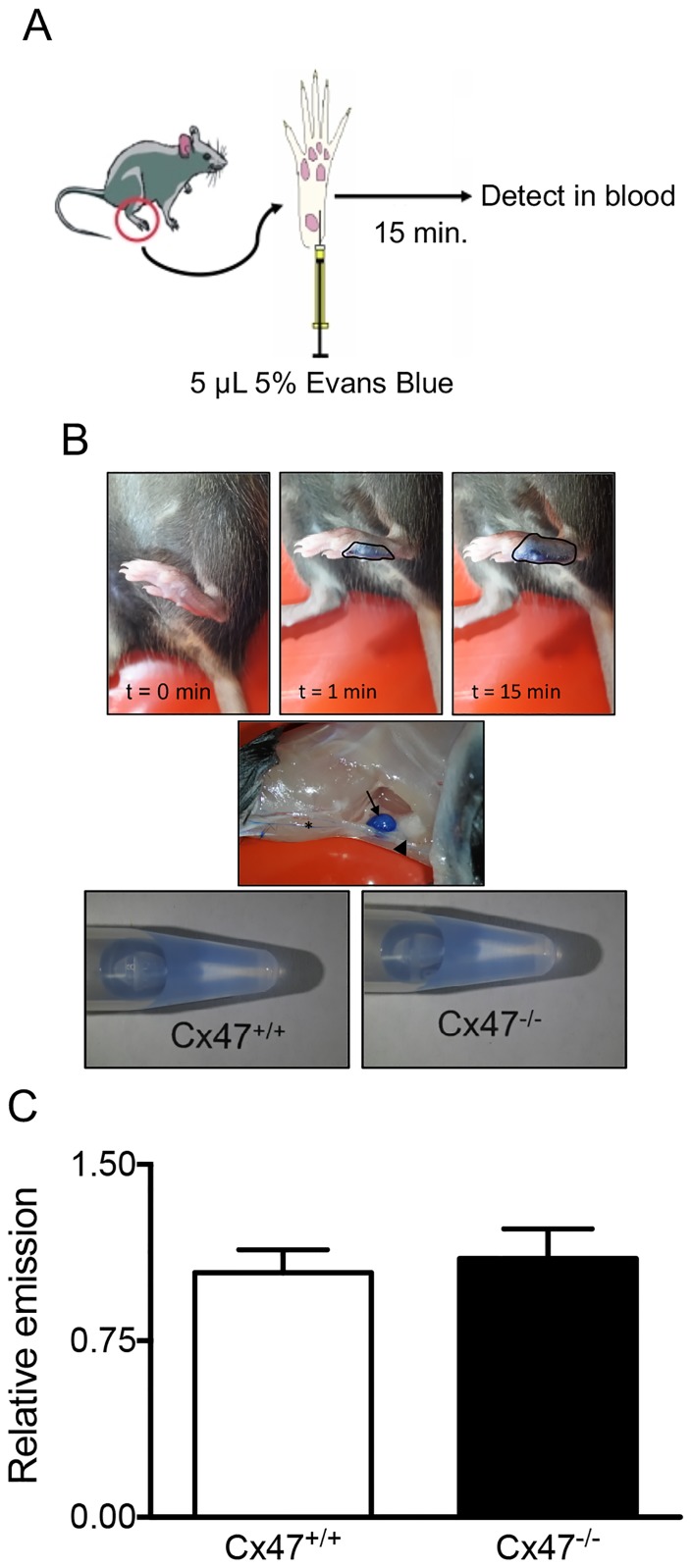
Lymphatic Cx47 does not regulate tissue drainage. A) Scheme illustrating the experimental procedure; mice received an Evans Blue injection in their footpad. Fifteen minutes after the injection, blood was collected and Evans Blue was detected in the serum using fluorescence. B) Typical examples of the spread of Evans Blue in the footpad of a *Cx47*^*+/+*^ mouse and examples of samples obtained from the serum of *Cx47*^*+/+*^ or *Cx47*^*-/-*^ mice. Initially (t = 0, t = 1; top images), the Evans Blue does not spread extensively (black line) from the site of injection. However, 15 minutes later, the spread is more extensive (black line). Moreover, 15 minutes after injection (middle image), the Evans Blue has spread towards draining lymph nodes (arrow) via afferent lymphatics (asterisk) and has advanced further via efferent lymphatics (arrowhead). Indeed, after 15 minutes (lower images) Evans Blue can readily be detected in the serum of *Cx47*^*-/-*^ (left image) and *Cx47*^*+/+*^ mice (right image). C) Quantification of the Evans Blue present in the serum of *Cx47*^*+/+*^ (white bar) or *Cx47*^*-/-*^ (black bar) mice 15 minutes after injection. N = 10/14.

It has been shown that gap junctions are involved in contractile lymphangion responses that regulate interstitial fluid drainage [20, 21]. In order to study tissue drainage, we quantified spread of Evans Blue towards the blood after injection in the left food pad (Fig 4A and [23]) of *Cx47*^*-/-*^ or *Cx47*^*+/+*^ mice. We clearly observed dermal spread of Evans Blue (Fig 4B) but Cx47-deficiency did not alter the extent to which Evans Blue spread towards the blood 15 minutes after injection (Fig 4C).

### Lymphatic Cx47 does not regulate lymphocyte trafficking

**Fig 5:**
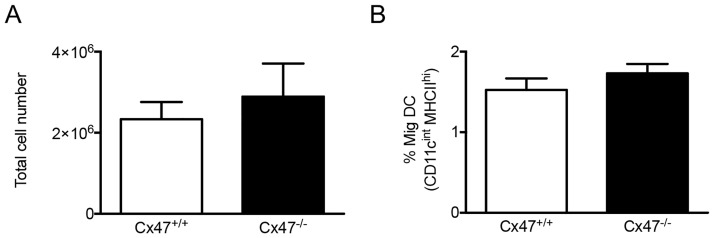
Cx47 does not modulate acute immune responses. A 1:1 mixture of acetone and dibutyl phthalate was applied epicutaneously to *Cx47*^*+/+*^ or *Cx47*^*-/-*^ mice. Twenty-four hours later, draining lymph nodes were excised and analyzed by flow cytometry using antibodies targeting CD11c or MHCII. In this classical model, Cx47-deficiency does neither affect A) the total amount of cells in inflammation draining lymph nodes nor B) the amount of migratory DCs in *Cx47*^*+/+*^ (white bars) or *Cx47*^*-/-*^ (black bars) mice. N = 6.

Lymphatic vessels are important regulators of immune responses; *i*.*e*. they form conduits by which immune cells can travel from sites of inflammation towards lymph nodes. It should be noted that immune cells within lymphatic vessels do not passively follow lymphatic flow. Rather, both dendritic cells (DCs) and T cells closely interact with LECs when they enter into lymphatic capillaries and then actively crawl from these capillaries into the downstream collecting vessels [24, 25]. Furthermore, LECs can present antigens and shape peripheral lymphocyte responses [26]. Moreover, gap junctions are involved in DC-mediated T lymphocyte activation [27]. Theoretically, Cx47 could play a role in any of these aspects regulating immunity. Thus, we studied migration of DCs in *Cx47*^*-/-*^ and *Cx47*^*+/+*^ mice using a model to induce lymphocyte trafficking. In line with earlier findings [28], exposure of the skin to a 1:1 acetone-butyl phthalate mixture resulted in enhanced DC migration towards draining lymph nodes. However, the total cell number in draining lymph nodes was not affected by absence of Cx47 (Fig 5A) and, importantly, the amount of migratory DCs in draining lymph nodes was not affected by Cx47-deficiency either (Fig 5B). Thus, Cx47 does not seem to modulate trafficking of DCs through the lymphatic system.

### Cx47 affects serum lipid handling but not uptake of long-chain fatty acids from the gut

**Fig 6:**
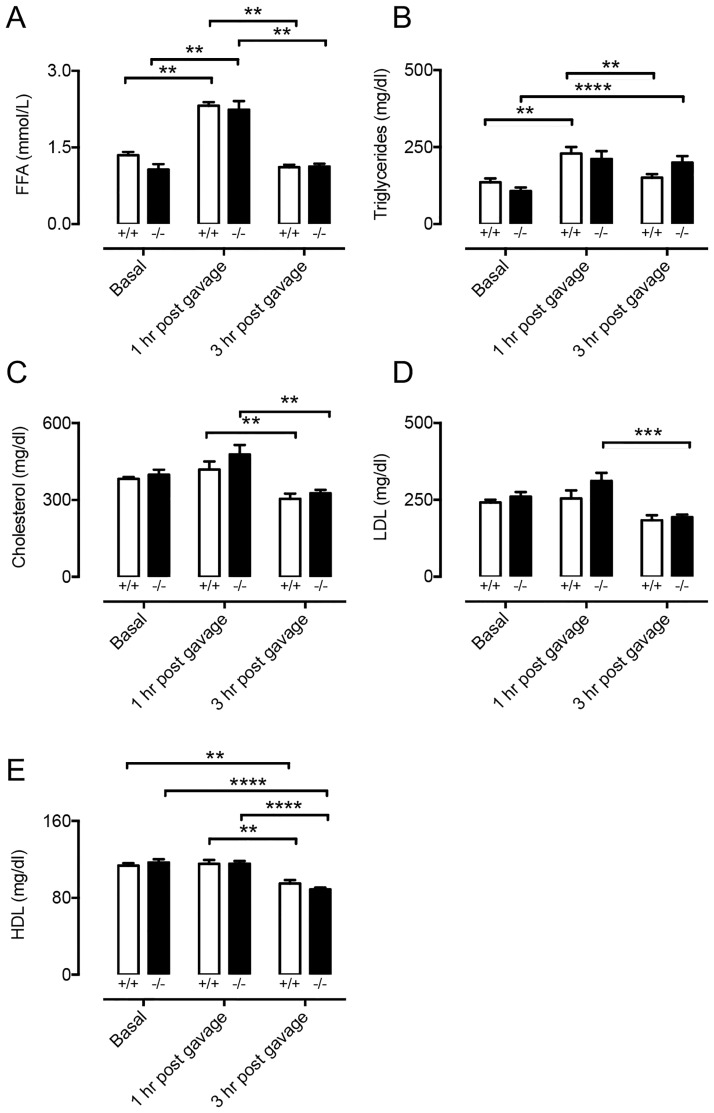
Uptake of long-chain fatty acids is not altered by Cx47-deficiency. A-E) Quantification of FFA (A), triglycerides (B), total cholesterol (C), LDL (D), or HDL (E) in serum of *Cx47*^*+/+*^ (white bars) or *Cx47*^*-/-*^ (black bars) mice at 0, 1 or 3 hours after oral gavage of olive oil (10 μl/gr bodyweight). Note that as expected, the serum content of FFA and triglycerides significantly increases whereas the level of total cholesterol, LDL and HDL is not affected by gavage. N = 6.

Lacteals, a special type of lymphatic vessels present in the gut, are vital for uptake of long-chain fatty acids from the intestine [29]. To investigate the role of Cx47 in lacteal function we performed oral lipid tolerance tests using *Cx47*^*-/-*^ and *Cx47*^*+/+*^ mice. As expected [29], we noticed an increase in both free fatty acids (FFAs) and triglycerides in the serum of *Cx47*^*+/+*^ mice 1 hour after gavage with olive oil (Fig 6A and 6B). Moreover, the FFA and triglyceride serum levels returned to basal values 3 hours after gavage (Fig 6A and 6B). As expected, exposure to olive oil did not affect serum cholesterol, low-density lipoproteins (LDL) or high-density lipoproteins (HDL) 1 hour post-gavage (Fig 6C–6E), however, their values tended to be decreased, or were significantly decreased 3 hours post-gavage. In *Cx47*^*-/-*^ mice exposure to olive oil had very similar effects: it resulted in a transient increase in FFA levels, and had no effect on cholesterol, HDL or LDL or tended to decrease presence of these lipids in the serum (Fig 6A and 6C–6E). However, serum triglyceride levels in *Cx47*^*-/-*^ mice remained increased until 3 hours after gavage (Fig 6B) whereas at this time point they had returned to baseline in *Cx47*^*+/+*^ mice. Thus, acute uptake of lipids from the intestine is not affected by Cx47-deficiency but serum lipid handling might be subtly regulated by Cx47.

### Cx47-deficiency affects serum lipid levels but not plaque size of chow-fed *Apoe*^*-/-*^ mice

**Fig 7:**
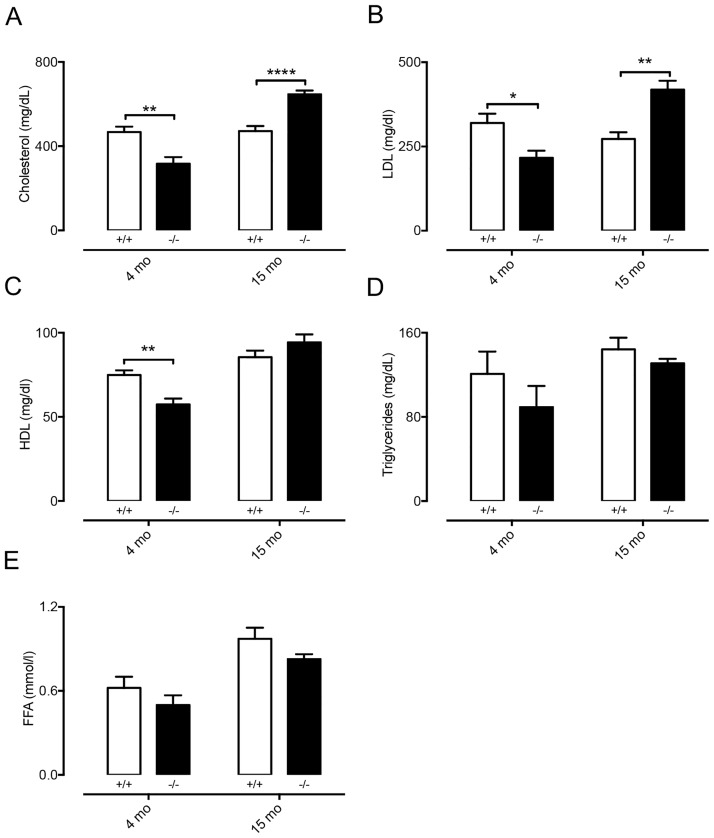
Serum lipid levels are affected by Cx47-deficiency. Serum lipids were determined in non-fasted 4 or 15 months-old *Cx47*^*+/+*^*Apoe*^*-/-*^ (white bars) or *Cx47*^*-/-*^ *Apoe*^*-/-*^ (black bars) mice. A) Total cholesterol levels are decreased in 4 month-old *Cx47*^*-/-*^ mice but increased in 15 month-old *Cx47*^*-/-*^*Apoe*^*-/-*^ mice. B) LDL serum levels are decreased in 4 month-old *Cx47*^*-/-*^*Apoe*^*-/-*^ mice but increased in 15 month-old *Cx47*^*-/-*^*Apoe*^*-/-*^ mice. C) HDL levels are decreased in in 4 month-old *Cx47*^*-/-*^*Apoe*^*-/-*^ mice. D-E) Cx47-deficiency does not alter serum free triglyceride (D) or fatty acid (FFA, E) in any experimental group. N = 6/11.

**Fig 8:**
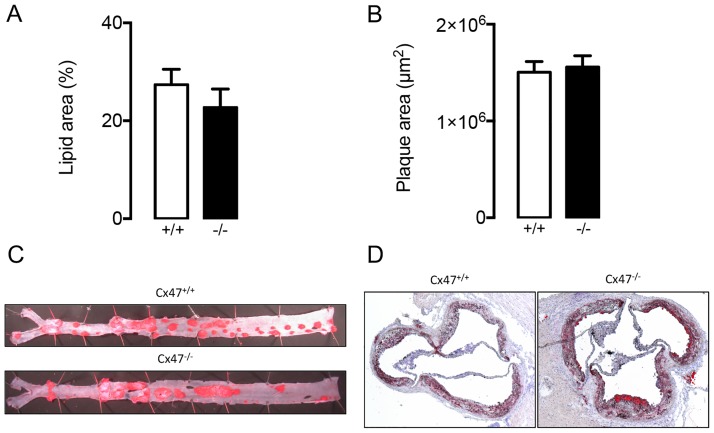
Spontaneous atherosclerotic plaque formation is not affected by Cx47-deficiency. A/B) Quantification of plaque size in thoracic-abdominal aortas (A) or aortic roots (B) as observed in 15 month-old *Cx47*^*+/+*^ *Apoe*^*-/-*^ (white bars) and *Cx47*^*-/-*^*Apoe*^*-/-*^ (black bars) mice. C/D) Representative images of C) *en face* thoracic-abdominal aortas or D) aortic roots stained with Sudan IV. N = 11.

There is increasing evidence for a role of lymphatics in atherosclerosis [30], a chronic inflammatory disease of the arterial wall in which serum lipids play a crucial role [31]. Since our results suggest that lipid handling is affected by Cx47, we first studied serum lipid levels in atherosclerosis-susceptible *Cx47*^*-/-*^ *Apoe*^*-/-*^ and *Cx47*^*+/+*^*Apoe*^*-/-*^ mice fed a regular chow diet. We initially compared serum lipid values in *Cx47*^*-/-*^*Apoe*^*-/-*^ of young adult or old mice (4 or 15 months) to serum lipid values in age-matched *Cx47*^*+/+*^*Apoe*^*-/-*^ mice. In 4 month-old *Cx47*^*-/-*^*Apoe*^*-/-*^ mice, we detected reduced levels of serum cholesterol, LDL and HDL as compared to *Cx47*^*+/+*^*Apoe*^*-/-*^ mice (Fig 7A–7C) whereas triglycerides and FFA were similar between genotypes (Fig 7D and 7E). In 15 months-old mice, the situation was partly reversed; *i*.*e*. we observed increased serum cholesterol and LDL levels in *Cx47*^*-/-*^*Apoe*^*-/-*^ mice as compared to *Cx47*^*+/+*^*Apoe*^*-/-*^ mice (Fig 7A and 7B). In this age group serum HDL, triglycerides and FFA were not affected by genotype (Fig 7C–7E). We next studied the spontaneous development of advanced atherosclerosis in 15 months-old *Cx47*^*-/-*^*Apoe*^*-/-*^ and *Cx47*^*+/+*^*Apoe*^*-/-*^ mice. Surprisingly, we observed that plaque size in thoracic-abdominal aortas (Fig 8A) or aortic roots (Fig 8B) was not affected by genotype despite altered serum lipid levels in *Cx47*^*-/-*^*Apoe*^*-/-*^ mice.

### Cx47-deficiency does not directly affect the size of diet-induced atherosclerotic plaques

**Fig 9:**
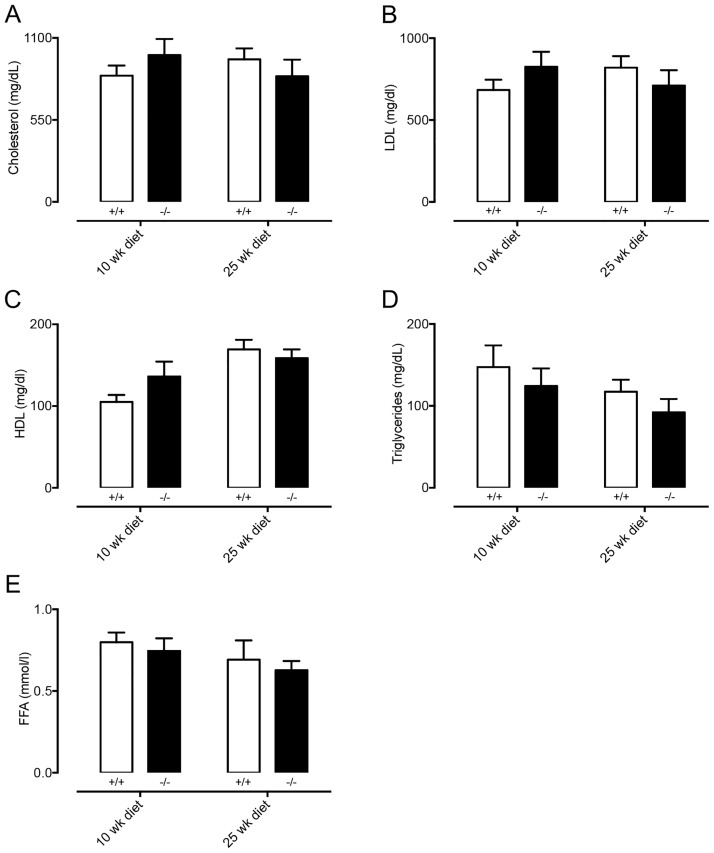
After high-cholesterol diet, serum lipid levels are unaffected by Cx47-deficiency. Serum lipids were determined in non-fasted *Cx47*^*+/+*^*Apoe*^*-/-*^ (white bars) and *Cx47*^*-/-*^*Apoe*^*-/-*^ (black bars) mice, which received high cholesterol diet for 10 or 25 weeks. In this experimental setting, serum cholesterol (A), LDL (B), HDL (C), triglycerides (D) or FFA levels are unaffected by genotype. N = 9/11.

**Fig 10:**
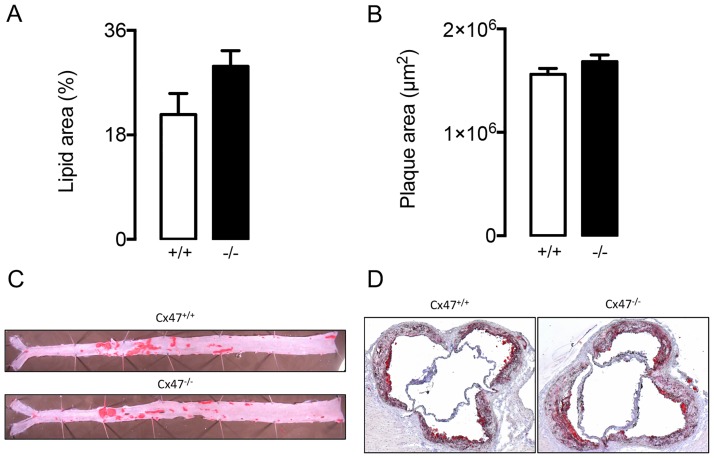
After high-cholesterol diet, atherosclerotic plaque formation tends to be enhanced by Cx47-deficiency. A/B) Quantification of plaque size in thoracic-abdominal aortas (A) or aortic roots (B) as observed in *Cx47*^*+/+*^*Apoe*^*-/-*^ (white bars) or *Cx47*^*-/-*^*Apoe*^*-/-*^ (black bars) mice. C/D) Representative images of C) *en face* thoracic-abdominal aortas or D) aortic roots stained with Sudan IV. N = 9/11.

Atherosclerotic plaque formation typically follows serum LDL-cholesterol values. Hence, since LDL-cholesterol was decreased in young adult *Cx47*^*-/-*^*Apoe*^*-/-*^ mice but increased in old *Cx47*^*-/-*^*Apoe*^*-/-*^ mice, we fed *Cx47*^*-/-*^*Apoe*^*-/-*^ and *Cx47*^*+/+*^*Apoe*^*-/-*^ mice from the age of 10-weeks a high-cholesterol diet for 10 or 25 weeks. After either 10 or 25 weeks of high-cholesterol diet, serum total cholesterol, LDL and HDL were elevated by 2-3-fold but similar for either genotype (Fig 9A–9C). Similarly, triglyceride and FFA levels were also not affected by the genotype (Fig 9D and 9E). As lymphatic vessels mostly arise in the vicinity of plaque hypoxia due to local VEGF-C/D release in advanced atherosclerotic plaques [30, 32, 33], we then studied plaque size in thoracic-abdominal aortas and aortic roots of *Cx47*^*-/-*^*Apoe*^*-/-*^ and *Cx47*^*+/+*^*Apoe*^*-/-*^ mice that had received cholesterol-rich diet for 25 weeks. These studies showed a trend towards increased plaque size in thoracic-abdominal aortas of *Cx47*^-/-^*Apoe*^*-/-*^ mice (Fig 10A, p = 0.07). Conversely, no genotype-induced differences were observed however in plaque size in aortic roots (Fig 10B).

### Plaque composition is subtly regulated by Cx47

**Fig 11:**
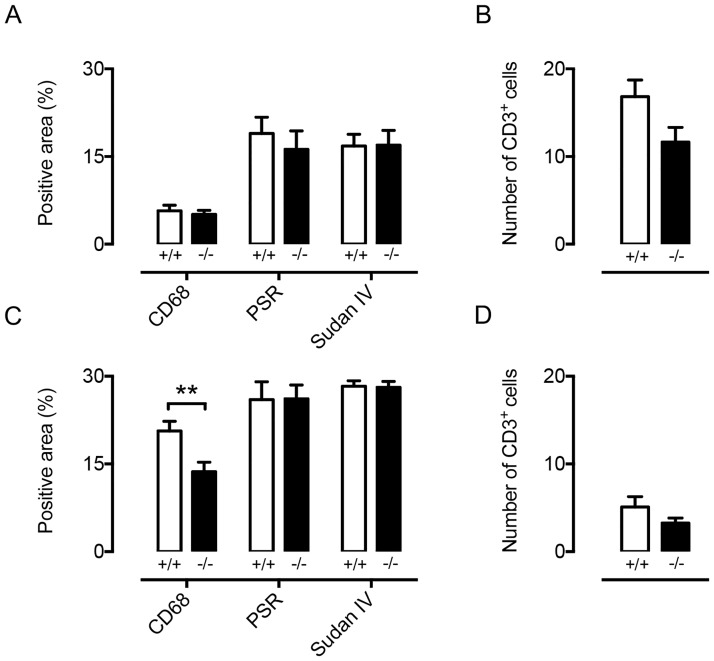
Cx47 subtly regulates atherosclerotic plaque composition. Plaque composition was determined in the aortic roots of 15 month-old *Cx47*^*+/+*^*Apoe*^*-/-*^ (white bars) and *Cx47*^*-/-*^*Apoe*^*-/-*^ (black bars) mice (upper panels) or *Cx47*^*+/+*^*Apoe*^*-/-*^ (white bars) and *Cx47*^*-/-*^*Apoe*^*-/-*^ (black bars) mice that had received high-cholesterol diet for 25 weeks (lower panels). A/B) In 15 month-old mice no differences between the genotypes were observed. C) In plaques from *Cx47*^*-/-*^*Apoe*^*-/-*^ (black bars) mice exposed to high cholesterol diet macrophage content was reduced whereas collagen and lipid content was unaffected by genotype. D) After a high-cholesterol diet T lymphocyte content was not affected by genotype. N = 9/11.

Atherosclerotic plaque size is not *per se* the most important determinant of disease severity in atherosclerosis. Indeed, patients presenting stable plaques (containing a low amount of macrophages, many smooth muscle cells and displaying a large lipid core and a relatively thick fibrous cap) are at lower risk for plaque rupture and thrombus formation as compared to patients with an unstable plaque [34, 35]. This is especially of interest in the context of lymphatic vessels in the vicinity of the plaque since these are important regulators of reverse cholesterol transport and immune cell migration [36–38]. Thus, we studied plaque composition in i) 15 month-old *Cx47*^*-/-*^*Apoe*^*-/-*^ mice in which serum LDL-cholesterol levels were increased compared with *Cx47*^*+/+*^*Apoe*^*-/-*^ controls and ii) in *Cx47*^-/-^*Apoe*^*-/-*^ mice that had received cholesterol-rich diet for 25 weeks and had elevated LDL-cholesterol levels in both genotypes. We focused on main determinants of plaque stability being: i) macrophages, ii) T lymphocytes, iii) collagen and iv) lipid content. In spontaneously developed advanced plaques in 15 months-old mice, we did not observe genotype-dependent differences regarding plaque macrophage, T lymphocyte, collagen or lipid content (Fig 11A and 11B). However, in diet-induced atherosclerotic lesions in *Cx47*^*-/-*^*Apoe*^*-/-*^ mice macrophage content was significantly reduced although collagen, lipid or T lymphocyte contents were not affected by genotype (Fig 11C and 11D).

## Discussion

LECs express *Cx47* ([10–13] and this study) and *GJC2* mutations are associated with lymphedema (3, 4). However, only sparse and fractional information about a role for Cx47 in lymphatic physiology exists. Hence, in the current study we thoroughly assessed involvement of Cx47 in lymphatic physiology. Specifically, we observed expression of Cx47 in LECs but not BECs from different mouse organs. Thereafter, we used genetic approaches to address the *in vivo* function of Cx47 regarding i) lymphatic morphology, ii) lymphatic pumping, iii) drainage of interstitial fluids, iv) acute immune responses in terms of migration of DCs towards antigen draining lymph nodes, v) uptake of long-chain fatty acids from the gut, vi) effects on serum lipid values during chow or high-cholesterol diet and vii) chronic immune responses in terms of modulation of atherosclerotic plaque size but also plaque content.

Our main findings are that i) Cx47 is involved in the reversal of acute increases in serum triglycerides, ii) serum lipid levels of mice on chow but not high-cholesterol diet are affected by Cx47 and iii) Cx47-deficiency tends to increase plaque size but decreases macrophage content of diet-induced atherosclerotic plaques.

Lymphatics, most notably lacteals, are critically involved in uptake of lipids from the intestine. Indeed, disrupted lacteal biology leads to disturbed serum lipid levels [29]. Moreover, lymphatic connexins, such as Cx37, have been shown to affect lacteal development [39] but whether this is also the case for Cx47 is unknown. Furthermore, whether Cx47 in LECs regulates lipid uptake is currently unclear. We showed that the uptake of FFA or triglycerides from the intestine is not affected by Cx47-deficiency (Fig 6). However, the data also show that dampening of the triglyceride peak, which occurred 1 hour after olive oil gavage, tended to be delayed in *Cx47*^*-/-*^ mice. These observations suggest that Cx47 may be fine-tuning the processing of serum lipids. This hypothesis was supported by the fact that in *Apoe*^*-/-*^ mice fed chow diet Cx47-deficiency resulted in enhanced or reduced serum lipid levels, depending on the age of the mice (Fig 7). This effect of Cx47 was no longer observed in *Apoe*^*-/-*^ mice fed a high-cholesterol diet, which is most likely due to a saturation of the lipid handling system (Fig 9). The mechanism by which Cx47 fine-tunes the handling of serum lipids is at present still not clear, but it should be noted that deletion or reduction of other connexins in the blood vessel or lymphatic endothelium (Cx37, Cx40 or Cx43) does not alter serum lipid levels [40–42]. Hence, it is unlikely that interactions between these connexins and Cx47 play a role. Moreover, Cx47 expression has not been reported in the main lipid handling organs such as adipose tissue, liver and skeletal muscle. Thus, a direct effect of Cx47-deficiency on lipid handling mediated by these organs seems also unlikely. In contrast, Cx47 is highly expressed in oligodendrocytes within the CNS [43, 44]. Importantly, it has been shown that the CNS can contribute to the regulation of serum lipids by controlling e.g. hepatic activity [45–47]. However, whether-or-not Cx47 in the CNS contributes to regulation of serum lipid metabolism is beyond the scope of this study.

Atherosclerosis is a chronic inflammatory disease of large- to medium-sized arteries. The disease is characterized by plaques that develop in the intimal space in arterial segments that are exposed to low and/or oscillatory shear stress [35, 48]. Advanced plaques contain lipids (most notably oxidized LDL), immune cells (most notably macrophages), extracellular matrix components (collagen) and newly developed (lymphatic) vessels. Classically, plaque immune cells have gained attention because of their role in maintenance of a persistent pro-inflammatory loop. Extracellular matrix components on the other hand have been studied since these structures provide much needed stability to plaques. Finally, newly formed lymphatic vessels have recently been suggested to play an important role in atherosclerotic plaques as well [30, 32, 33]; *i*.*e*. they provide pathways by which immune cells (e.g. macrophages) can egress from the plaques thereby facilitating reverse cholesterol transport [36] and are thus thought to be anti-atherogenic. In addition, the lymphatic system can also affect atherogenesis by modulating serum lipid values which generally directly translate into enhanced, or reduced, atherosclerotic plaque burden [31]. Hence, one would expect enhanced atherogenesis in mice characterized by increased serum total cholesterol or LDL levels (e.g. in 15 months-old Cx47-deficient mice) and decreased atherogenesis in mice with decreased serum levels of these lipids (such as the 4 months-old Cx47-deficient mice). However, we found a tendency to increased plaque development in thoracic-abdominal aortas of Cx47-deficient mice that had received high-cholesterol diet for 25 weeks (Fig 10A). This difference in plaque development was not induced by serum lipid levels, because those were similar in Cx47-expressing and Cx47-deficient mice on a high-cholesterol diet both after 10 weeks and 25 weeks of diet. Thus, lymphatic Cx47 could be reducing plaque size in thoracic-abdominal aortas of *Cx47*^*+/+*^*Apoe*^*-/-*^ mice by enhancing egress of immune cells. Intraplaque neovessels are generally thought to arise from preexisting adventitial structures [30, 32, 33]. Hence, one would expect that a possible anti-atherogenic role of Cx47 would not be found in atherosclerotic plaques at locations characterized by low levels of adventitial lymphatics such as aortic roots. Our observations with respect to plaque development in the aortic roots of *Cx47*^*-/-*^*Apoe*^*-/-*^ and *Cx47*^*+/+*^*Apoe*^*-/-*^ mice confirm this hypothesis (Fig 10B).

The amount of plaque macrophages was slightly reduced in *Cx47*^*-/-*^*Apoe*^*-/-*^ mice that had received high-cholesterol diet for 25 weeks (Fig 11C), suggesting enhanced egression of macrophages from atherosclerotic plaques in Cx47-deficient mice. Egression of immune cells from atherosclerotic plaques might be affected by changes in lymphatic contractility, lymphatic-mediated interstitial tissue drainage and/or lymphatic-dependent immune cell migration. However, our findings suggest that Cx47-deficiency is not affecting any of these processes directly (Figs 3–5). Finally, macrophages do not express Cx47. Hence, a direct effect of Cx47-deficiency on processes such as macrophage proliferation/apoptosis/necrosis seems unlikely as well.

Altogether, our data show that Cx47-deficient mice do not display an overt lymphatic phenotype. This is surprising since mutations in GJC2 have been linked to primary and secondary lymphedema in patients [3, 4]. The reason(s) for the apparent discrepancy between the findings in patients and our findings in mice are not entirely clear. However, it should be kept in mind that several connexins (most notably, Cx37, Cx43 and Cx47 [9–11]) are co-expressed in lymphatic endothelium. A first explanation may be that the level of endogenous Cx37/Cx43 expression by itself is sufficient to maintain adequate connexin function in absence of Cx47. Secondly, knockout mice, or even mouse models with the same causative genetic mutations, do often not fully recapitulate human disease or sometimes fail to show any phenotypic alteration, suggesting the existence of compensatory mechanisms [49]. Thus, a compensatory up-regulation of Cx43 for example might have counterbalanced for the loss of Cx47 in our mouse models. This would by itself not be entirely surprising since compensatory mechanisms in global (connexin) gene knockouts have been observed before [50]. One should however keep in mind that functional replacement of one connexin gene with another in mice has demonstrated that cellular homeostasis does not only depend on the presence of intercellular communication through gap junction channels but also requires the correct type of connexin in the target cell [51] or even the correct type of connexin in adjacent cells. Indeed, Cx47 phosphorylation and stability in oligodendrocytes has been shown to critically depend on Cx43 expression in astrocytes, suggesting that there may be an intricate interplay between these two connexins [52]. Finally, one should keep in mind that different connexin isoforms (i.e. Cx47, Cx37, Cx43) may assemble in various combinations into heteromeric connexons (hemi-channels) and that differently assembled connexons can dock into heterotypic gap junction channels [53–55]. Consequently, mutations in connexin genes can give rise to mutant forms of the proteins that can have dominant negative effects on other connexins within the same hemi-channel or gap junction channel [3, 56]. For example, the Cx47-R260C mutation located within the SRPTEK motif in the second extracellular loop may have such dominant negative effects. This motif is important for connexon docking and a target for mimetic peptides that inhibit gap junctional communication [57–59]. Thus, lymphedema-associated Cx47 mutations in patients may not result in a loss-of-function of the Cx47 protein but may rather result in a dominant negative effect on Cx43, another connexin expressed in LECs, thereby resulting in altered function of heteromeric/heterotypic gap junctions affecting lymphatic physiology. Although knock-out technologies have taught us much on the *in vivo* physiological role of many important proteins, use of genome editing approaches is necessary for analysis of dominant negative phenotypes.

## Materials and methods

### Animals

Animal experimentation conformed to the *Guide for the Care and Use of Laboratory Animals* published by the US National Institutes of Health and was approved by Swiss cantonal veterinary authorities. Atherosclerosis-susceptible mice with deletion of Cx47, *i*.*e*. *Cx47*^*-/-*^*Apoe*^*-/-*^ mice, on a C57BL/6 background were generated by crossing *Apoe*^*-/-*^ mice [60] and mice expressing eGFP controlled by the Cx47 promoter (Cx47^eGFP/eGFP^*)* [16]. In addition, *Cx47*^*-/-*^ and *Cx47*^*+/+*^ mice were crossed with *Prox1*^*tg/+* mice^ [61] to visualize lymphatic vasculature in Cx47-expressing and Cx47-deficient mice. Genotypes of the different mice were verified by PCR using previously described protocols [16, 61]. Mice were in conventional housing on a 12-hour night-day cycle and had free access to food (either high-cholesterol diet or chow pellets).

### Quantitative PCRs on organs

Mice were killed by cervical dislocation. Thereafter, organs were collected and snap frozen. Subsequently, RNA was isolated and qPCRs were performed using primers (Thermo scientific) as previously described [62].

### Isolation of LECs and BECs

LECs and BECs were isolated using cell sorting as previously described [29]. Total RNA was isolated using the QIAGEN RNeasy Plus Micro Kit. Reverse transcription was performed using Transcriptor First Strand cDNA Synthesis Kit (Roche Diagnostics). Alternatively, mRNA was amplified using Ovation Pico WTA System V2 (NuGEN). qPCRs were performed using a StepOnePlus apparatus (Applied Biosystems) using SYBR Fast PCR Master Mixes (Kapa Biosystems). Analysis of gene expression was carried out using the comparative Ct (ΔΔCt) method as described by the manufacturer. Sequences of PCR primers are provided in S1 Table.

### Visualization of eGFP in LECs

eGFP was visualized in LECs as described before [17]. In brief, mice were culled and perfused with heparinised PBS via the aorta prior to fixation in 4% paraformaldehyde (PFA) followed by blocking in 3% v/v donkey serum in 0.3% Triton-x100. Samples were further dissected prior to incubation with primary antibodies, and washed prior to localization with fluorophore-conjugated secondary antibodies. Samples were then further dissected and mounted in Prolong Gold (Invitrogen). The lymphatics were visualized by confocal microscopy (Leica SP5) to produce Z projections (NIH ImageJ) of median filtered (Leica LASAF/ImageJ) stacks. The following antibodies were used: rabbit anti-Prox1 (11-002P, Angiobio), goat anti-GFP (ab6658, Abcam, Biotinylated), rat anti-PECAM1 (BD clone MEC 13.3) and mouse anti-alpha smooth muscle actin (clone 1A4 conjugated to Cy3, Sigma).

### Quantification of the number of lymphatic valves, lymphatic diameter and lymphatic contractility

*Cx47*^*-/-*^*Prox1*^*tg/+*^ or *Cx47*^*+/+*^*Prox1*^*tg/+*^ mice were sedated as previously described [29]. The diameter of the lymphatic collecting vessel draining the inguinal lymph node was measured by intravital microscopy using a Leica M205FA stereomicroscope and simultaneously video analysis was performed to quantify lymphatic contractility in terms of percentage of contraction and contractile frequency. Thereafter, mice were killed by cervical dislocation and the ears of the mice were isolated and processed. In brief, the ears were fixed overnight at 4°C and subsequently washed with PBS. Then the inner and outer parts of the dermis of the ear were separated using forceps. For the quantification of lymphatic valves, we focused on the inner part of the dermis. After separation, the inner part of the dermis was mounted on a microscopy slide within the wall of three Secure-Seal spacers (Molecular Probes) in order to maintain the three-dimensional structure. Thereafter, lymphatic vessels were visualized using a LSM700 confocal microscope (Zeiss). Three separate recordings were obtained in order to capture >75% of the total ear surface. Z-stacks covering the layer of tissue in which lymphatics are present were obtained during these recordings in order to capture all the lymphatics. After imaging the Z-stacks were converted to a single plain using Fiji using the Z Project plugin. The number of lymphatic valves was thereafter counted manually by two observers who were unaware of the genotype of the mice and was subsequently normalized for the area of the ear.

### Quantification of interstitial fluid drainage

Drainage of interstitial fluids was quantified using Evans Blue injections in the footpad of *Cx47*^*-/-*^*Apoe*^*-/-*^ or *Cx47*^*+/+*^*Apoe*^*-/-*^ mice as described previously [23]. In brief, mice were sedated as described above. Once anesthetized, the mice were injected with 5 μl 5% Evans Blue (dissolved in PBS) using a micro syringe. Subsequently, 15 minutes after the initial injection, blood was collected from the left ventricle and serum was obtained. The serum was then incubated overnight with formamide (Sigma-Aldrich; 1:1 ratio) at 55°C. Thereafter, presence of Evans Blue in the serum was quantified using a SpectraMax Paradigm Multi-Mode Microplate reader (Molecular Devices).

### Quantification of long-chain fatty acid uptake

The uptake of long-chain fatty acids was quantified as previously described [29]. In brief, *Cx47*^*-/-*^*Apoe*^*-/-*^ and *Cx47*^*+/+*^*Apoe*^*-/-*^ mice were subjected to an overnight fast and subsequently received olive oil (Sigma-Aldrich; 10 μl/gr bodyweight) by oral gavage. Blood was obtained by submandibular puncture and the amount of serum lipids was determined at t = 0, 1 and 3 hours after gavage using a Cobas C111 (Roche Diagnostics).

### Quantification of serum lipids and isolation of tissues to study atherogenesis

*Cx47*^*-/-*^*Apoe*^*-/-*^ and *Cx47*^*+/+*^*Apoe*^*-/-*^ mice were used. They either received a high cholesterol diet (1.5%, 0% cholate; Research Diets) for 10 or 25 weeks starting from the age of 10 weeks or, alternatively, were fed regular chow until an age of 4 or 15 months. At the experimental day, the mice were sedated as described above and blood was collected from the left ventricle, serum was isolated and serum lipids were determined using a Cobas C111 (Roche Diagnostics). After collection of the blood, the aortas of the mice were perfused with 0.9% NaCl before being separated in the thoracic-abdominal part, which was fixed overnight in 2% PFA, and the aortic root, which was frozen in OCT compound.

### Quantification of plaque size

The extent of atherosclerosis in the thoracic-abdominal aortas and the aortic roots was quantified according to standard protocols [41, 63]. In brief, the thoracic-abdominal were stained with Sudan IV and subsequently opened longitudinally and the extent of staining was assessed using Fiji software. Plaque size in the aortic roots (in μm^2^) was also determined using Fiji software in six 7-μm sections at 50 μm distension.

### Plaque content quantification

7-μm sections from the aortic roots were immunolabeled with antibodies against CD68 (AbD Serotec) or CD3 (DakoCytomation). Collagens were stained with Picrosirius Red and lipids were stained using Sudan IV. Macrophage, collagen and lipid content were quantified by dividing the CD68, Picrosirius Red or Sudan IV positive area by the total plaque area using Image J. The amount of plaque T lymphocytes was quantified by manual counting by two observers who were unaware of the genotype of the mice.

### Quantification of *in vivo* DC migration

*Cx47*^*-/-*^*Apoe*^*-/-*^ and *Cx47*^*+/+*^*Apoe*^*-/-*^ mice were used and dendritic cells migration was induced as described previously [28]. Briefly, a 1:1 mixture of acetone and dibutyl phthalate was applied epicutaneously. Twenty-four hours later, draining lymph nodes were excised and analyzed by flow cytometry using antibodies targeting CD11c (clone N418) and MHCII (clone AF6.120.1) using a FACSCalibur (BD). Analysis was performed using FlowJo software (Tree Star).

### Statistics

Results are shown as mean ± SEM. One-way ANOVA or two-way Student’s T-tests were performed by Graphpad Prism 6.0 to compare the means between the groups as indicated in the figure legends. Differences with a P < 0.05 were considered significant; *, P < 0.05; **, P < 0.01; ***, P < 0.001; ****, P < 0.0001.

## Supporting information

S1 Table Primers used for qPCR analysis. (PDF)

S1 Movie Typical example of lymphatic contractions in a *Cx47*^*+/+*^*Prox1*^*tg/+*^ mouse. (MOV)
