# Supplementary material for: Cx47 fine-tunes the handling of serum lipids but is dispensable for lymphatic vascular function
Source: PLoS One. 2017 Jul 21;12(7):e0181476. doi: 10.1371/journal.pone.0181476 (PMC5521787; doi:10.1371/journal.pone.0181476)
Supplement: S1 Table — (PDF) [file pone.0181476.s001.pdf]

Supplemental Table 1: **Primers used for qPCR analysis**

Gene	Species	Forward primer	Reverse primer
18S	Human/mouse	5'-AGGAATTCCCAGTAAGTGCG	5'-GCCTCACTAAACCATCCAA
Gjc2	Mouse	5’-TCCACAATCATTCCACCTTCG	5’-CAGAAGCGCACATGAGACA
Prox1	Mouse	5'-AAGATATGTCCGACATCTCACCTTATTCAG	5'-CACGTCCGAGAAGTAGGTCTTCAG
Flt1	Mouse	5'-TGGCTCTACGACCTTAGACTG	5'-CAGGTTTGACTTGTCTGAGGTT
